# A Deep Intronic HADH Splicing Mutation (c.636+471G>T) in a Congenital Hyperinsulinemic Hypoglycemia Case: Long Term Clinical Course

**DOI:** 10.4274/jcrpe.1963

**Published:** 2015-06-03

**Authors:** Emine Çamtosun, Sarah E. Flanagan, Sian Ellard, Zeynep Şıklar, Khalid Hussain, Pınar Kocaay, Merih Berberoğlu

**Affiliations:** 1 Ankara University Faculty of Medicine, Department of Pediatric Endocrinology, Ankara, Turkey; 2 Exeter University Faculty of Medicine, Institute of Biomedical and Clinical Science, Exeter, UK; 3 UCL Institute of Child Health, Genetics and Epigenetics in Health and Disease Genetics and Genomic Medicine Programme, London, UK; 4 Great Ormond Street Hospital for Children, Clinic of Pediatric Endocrinology, London, UK

**Keywords:** HADH mutation, hyperinsulinemic hypoglycemia, children

## Abstract

Unlike other congenital fatty acid oxidation defects, short-chain L-3-hydroxyacyl-CoA (SCHAD, HADH) deficiency is characterised by hypoglycemia with hyperinsulinism in the neonatal or infancy periods. The long-term and detailed clinical progression of the disease is largely unknown with almost 40 patients reported and only a few patients described clinically. We present clinical and laboratory findings together with the long-term clinical course of a case with a deep intronic HADH splicing mutation (c.636+471G>T) causing neonatal-onset hyperinsulinemic hypoglycemia with mild progression.

## INTRODUCTION

Persistent congenital hyperinsulinemic hypoglycemia (CHH) can be caused by mutations in the ABCC8/KCNJ11, GLUD1, HADH, GCK, HNF4A, HNF1A, SLC16A1 and UCP2 genes (1,2). ABCC8/KCNJ11 mutations are most common (33-66%) and GLUD1 mutations are the second commonest cause of CHH, identified in approximately 5% of the cases. Mutations in other known genes (including HADH) together account for less than 4% of CHH (1).

Loss of function mutations in the HADH (HADHSC, SCHAD) gene, causing short-chain L-3-hydroxyacyl-CoA (SCHAD) deficiency, were first reported to cause persistent CHH in 2001 (3). SCHAD catalyses the penultimate step in the mitochondrial fatty acid oxidation pathway, the NAD+-dependent conversion of L-3-hydroxyacyl-CoA to 3-ketoacyl-CoA (1). The main clinical feature of this metabolic disease is hypoketotic hypoglycemia with hyperinsulinism which is different from other inherited defects of fatty acid β-oxidation that can present with symptoms such as hepatomegaly, myopathy and cardiomyopathy. HADH mutations are recessively inherited and most of the reported cases are from consanguineous families. So far, approximately 40 patients with CHH resulting from a mutation in the HADH gene have been reported (Table 1) (3,4,5,6,7,8,9,10,11,12,13,14,15).The mechanism behind unregulated insulin secretion in SCHAD deficiency is currently not understood but may involve changes in protein-protein interactions with glutamate dehydrogenase (GDH) (16,17).

The clinical presentation is mainly neonatal- or early infancy-onset HH and patients are diazoxide-responsive. It has also been shown that these patients have severe protein (especially leucine) sensitivity (10,16,17). Metabolic profiling in some, but not all, affected individuals reveals a raised plasma hydroxybutyrylcarnitine and urinary medium-chain dicarboxylic, 3-hydroxydicarboxylic metabolites and 3-hydroxyglutarate levels.

Here we present the long-term follow-up of a case with a deep intronic HADH splicing mutation (c.636+471G>T) causing hyperinsulinemic hypoglycemia and review the reported cases so far.

## CASE REPORT

A 13.9-year-old girl was admitted to our hospital with complaints of headache and weakness. She had a convulsion attack with hypoglycemia one day before admission. Her medical history revealed that she had been diagnosed with persistent CHH at the age of 30 days following admission to a health center for generalised convulsion. She was born at term with a weight of 4000 g. Following the diagnosis of CHH, she was commenced on 10 mg/kg/day of diazoxide and remained on that dose for five years.

At the age of 2 years, the patient was lost to follow-up for 5 years. Then, she presented at seven years of age with generalized convulsion with a hypoglycemic attack due to interrupted intake of medicine. After restarting diazoxide therapy (10 mg/kg/day) with regular intake, the girl did not have any hypoglycemic attacks but developed hypertrichosis.

At the age of 12.5 years, the patient was re-evaluated in another medical center. During hypoglycemia, her insulin level was detected to be very high (78.8 µIU/mL). Urinary amino acids and blood spot tandem mass were normal; there were no reducing substances in the urine. The results of a prolonged oral glucose tolerance test were normal. The diazoxide dose was tapered to 2.5 mg/kg/day slowly without episodes of hypoglycaemia. Drug cessation was tried but because of repeated hypoglycemic attacks diazoxide was started again at a dose of 2.5 mg/kg/day. When the girl was first admitted to our clinic after a hypoglycemic convulsion, she had complaints of headache and weakness, but physical examination was normal; height 153 cm (-0.93 SD) and weight 47 kg, body mass index (BMI) was 20.08 kg/m2. She was hospitalized for further evaluation. A fasting test was performed, but hypoglycemia was not detected during a 12-hour fast. While she was taking diazoxide 2 mg/kg/day, a non-ketotic hypoglycemic attack (blood glucose: 46 mg/dL) was detected with a very high insulin level (46.8 µIU/mL). During this episode, blood gas sampling, blood ammonia, cortisol and adrenocorticotropic hormone were all within the normal range.

**Figure 1:**
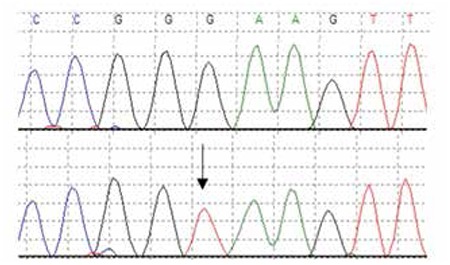
Electropherogram showing the homozygous c.636+471G>T cryptic splicing mutation in intron 5 of the HADH gene (lower panel). A sequence trace for a control (upper panel).

Cranial MRI and an electroencephalogram did not reveal any pathology. Normoglycemia was maintained with diazoxide 3 mg/kg/day. Genetic studies by Sanger sequencing did not reveal any mutations in the ABCC8, KCNJ11 or GLUD1 genes, however, sequencing of HADH identified a previously reported homozygous splicing mutation within intron 5 (c.636+471G>T) (Figure 1) (10). The parents who are not known to be consanguineous were both heterozygous for the mutation. The patient is currently 20 years of age and in good health. Mental status and neurological examination were normal at her last follow-up visit. She was reported to have maintained her academic performance at the higher education institution she was attending. Her blood glucose levels continued to be at normal levels with the low dose (2-3 mg/kg/day) of diazoxide she has been receiving. She has reached her final height which is within normal ranges and has a BMI of 24.8 kg/m2.

Informed consent was given by the patient.

## DISCUSSION

Recessive mutations in the HADH gene were first described by Clayton et al (3) in a four-month-old infant with hypoketotic hypoglycemia, inappropriately elevated insulin levels and elevated blood spot hydroxybutyrylcarnitine concentration. Since then, another 36 patients have been reported with HADH mutations (4,5,6,7,8,9,10,11,12,13,14). The clinical presentation of CHH due to HADH mutations is heterogeneous with some patients presenting with severe neonatal hypoglycemia and others - with mild infancy-onset hypoglycemia. Our case was born at term with a weight of 4000 g (1.63 SD) and presented with neonatal hypoglycemic seizure at the age of 30 days. Birth weight of patients with HADH gene mutation were usually reported as normal from 2730 g to 4350 g (3,4,5,6,7,8,9,10,11,12,13,14,15). Early admitted patients usually had hypoglycemic seizure as our case (3,4,5,8,9,10,13,14,15). Martins et al (4) reported four new cases and reviewed seven reported cases with HH due to HADH deficiency. They found that patients became symptomatic in early life (ranging from 1.5 hours to 8 months) and presented with hypoglycemic convulsions, lethargy and hypotonia. Although their symptoms had begun in infancy, three of the four new cases were diagnosed late and presented with mental retardation, microcephaly. Flanagan et al (5) reported 11 cases with a median age of 7 weeks (1 day-26 weeks) at HH diagnosis and a median birth weight of 3.6 kg (2.8-4.35 kg) at 40 weeks gestation When Kapoor et al (6) studied the clinical and molecular characterisation of 300 patients with CHH, they identified three patients who had HADH mutations and were diazoxide-responsive. Mean birth weight was -1.08 SD and mean age at presentation was 125 days in these patients (6). Snider et al (7) investigated genotype and phenotype correlations in 417 children with CH and reported two diazoxide-responsive patients with HADH gene mutations. Clinical information was not provided for these patients. Flanagan et al (11) identified eight new diazoxide-responsive CHH patients with a deep intronic splicing mutation in the HADH gene by next-generation sequencing.

All cases reported in the literature are diazoxide-responsive with a range in dose used from to 2-15 mg/kg/day (median 7-8 mg/kg/day) (4,5). Comparable with other patients reported in the literature, our case was diazoxide-responsive with 10 mg/kg/day at the beginning and the maintenance dose at older ages was 2.5-3 mg/kg/day. To identify and treat HH earlier is crucial for preventing persistent brain damage. Even for diazoxide-responsive patients, if not treated properly, the outcome can be dramatic like motor mental retardation, cortical blindness, microcephaly, etc. Due to the early diagnosis and proper treatment, our patient’s prognosis was very good.

The metabolic profile is heterogeneous in patients with HADH mutations. The first three reported patients had detectable urinary 3-hydroxyglutarate and raised plasma 3-hydroxybutyryl-carnitine levels; subsequently reported cases however did not have abnormal urine organic acids or acylcarnitines (5). We could not analyse the urine organic acid profile of our patient.

Kapoor et al (10) reported a patient with CHH due to a HADH gene mutation, who responded to diazoxide but continued to have episodes of hypoglycemia even on diazoxide especially when taking a meal rich in protein. The authors described protein sensitivity in this and two other cases (10). A study by Heslegrave et al (17) further supported this finding and showed that GDH and HADH have a direct protein-protein interaction which is lost in patients with HADH gene mutations causing leucine-induced HH. A protein-load test has not been undertaken in our patient, but after a protein-rich meal, blood glucose level was detected as 62 mg/dL. There is almost no information about clinical findings of these patients with the same mutation. Only limited clinical findings of two siblings with the same mutation was published as a short report. Both has been admitted to hospital at 40 days of age with hypoglycemia-related seizures. Birth weights were normal in these patients and they were successfully treated with diazoxide. There is no additional follow-up information (15).

Although consanguinity was not reported in our family, most cases in the literature are known to be consanguineous. As the disease is recessively inherited, sequencing of HADH is recommended in all patients with diazoxide-responsive CHH, following exclusion of mutations in ABCC8/KCNJ11 genes (1,5), who originate from known consanguineous pedigrees, isolated populations or countries where inbreeding is frequent.

So far, reported mutations in the HADH gene include missense, nonsense, frameshift and splicing mutations, some small deletions and a deep intronic splicing mutation (3,6,10). The mutation identified in our patient was a deep intronic splicing mutation which was first detected by targeted next-generation sequencing (11). This mutation has been shown to introduce a cryptic splice donor site which results in pseudoexon activation and a premature termination codon and was also a founder mutation in the Turkish population (11).

In the long-term follow-up period, our patient showed mild CHH. The prognosis was good with low-dose diazoxide treatment. The findings in our patient demonstrate that the recognition of hyperinsulinism as a cause of hypoglycemia in early life and early treatment can prevent neurological deficits especially in diazoxide-responsive forms of CHH like HADH deficiency.

## Figures and Tables

**Table 1:**
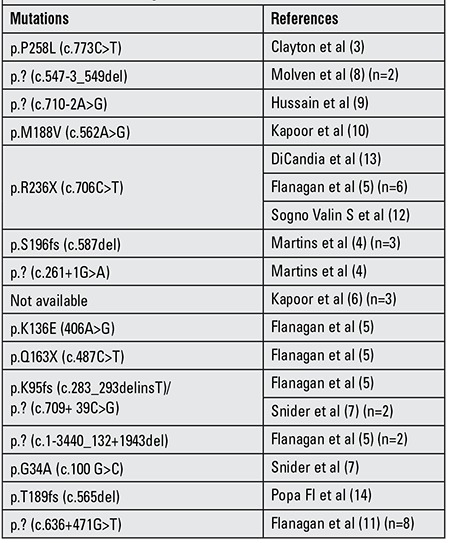
Reported patients with hyperinsulinemic hypoglycemia having mutations in the HADH gene.
